# The Mental Health Impact of 2019-nCOVID on Healthcare Workers From North-Eastern Piedmont, Italy. Focus on Burnout

**DOI:** 10.3389/fpubh.2021.667379

**Published:** 2021-05-11

**Authors:** Carla Gramaglia, Debora Marangon, Danila Azzolina, Chiara Guerriero, Luca Lorenzini, Manuela Probo, Marco Rudoni, Eleonora Gambaro, Patrizia Zeppegno

**Affiliations:** ^1^Department of Translational Medicine, Università del Piemonte Orientale, Vercelli, Italy; ^2^Psychiatry Unit, Maggiore della Carità Hospital, Novara, Italy; ^3^Department of Mental Health, ASL NOVARA, Novara, Italy

**Keywords:** COVID-19, burnout, anxiety, depression, healthcare worker

## Abstract

The 2019-nCOVID pandemic as a public health emergency has faced healthcare systems with unprecedented challenges. Our study aimed to focus on the mental health impact of the 2019-nCOVID pandemic on healthcare workers (HCWs) from North-Eastern Piedmont, Italy. For this purpose, we performed an online survey which was e-mailed to HCWs at the end of the first peak of the pandemic. We involved both frontline and not-frontline HCWs, employed in the hospital or in healthcare services outside the hospital. The primary outcome of our research was the assessment of burnout, while secondary outcomes included the investigation of anxiety, depression, and post-traumatic stress symptoms. We observed higher levels of burnout (especially in the Depersonalization and Personal Accomplishment dimensions), in females, in HCWs aged <30 years, in those exposed to changes in their daily and family habits, in those who had to change their duties at work and in residents in training. In our HCWs sample we found lower levels of anxiety and depression than those reported in the literature. The problematic levels of burnout and adverse psychological outcomes observed during the pandemic cannot be underestimated. Given the recurrence in autumn 2020 of a new pandemic peak, which has once again put a strain on the health system and HCWs, it is supported the importance of a careful assessment of HCWs' mental health, and of the possible risk and protective factors both in the work environment and in the extra-work one.

## Introduction

The 2019-nCOVID pandemic as a public health emergency has faced healthcare systems with unprecedented challenges. Especially at the beginning, the focus of efforts has been mainly on the necessary, “practical” issues, such as management of intensive care unit beds, availability of personal protective and medical equipment, while less attention has been paid to taking care of the psychological impact of the pandemic on healthcare workers (HCWs). HCWs were (and are) put under pressure and heavily burdened by the several challenges in the treatment of 2019-nCOVID patients, and in providing continuous support to non 2019-nCOVID ones. A further challenge of the current situation has been the need to find new ways to provide emotional and psychological support in a general condition of heightened levels of uncertainty, stress, fear and anxiety, with a high risk of burnout (1). Possible consequences of providing healthcare, either frontline or not, during viral epidemics may include general health concerns, fear, insomnia, somatization, stigmatization feelings, psychological distress, acute, and post-traumatic stress disorder, anxiety, burnout, depression (2–5). Some recent systematic reviews suggested that specific socio-demographic, individual, social, and occupational features might represent either protective or risk factors for these psychological outcomes (3, 5). With more detail, risk factors included being younger and female, working in a high-risk environment, lower levels of specialized training and job experience, lacking social support and stigmatizing experiences, neuroticism, maladaptive coping styles, poor resilience, and self-efficacy.

A recent systematic review and meta-analysis (6) 4/2/2021 10:34:00 PMincluded 13 studies with the aim to analyze the prevalence of depression, anxiety, and insomnia among HCWs during 2019-nCOVID pandemic. Anxiety was assessed by almost all studies (pooled prevalence of 23.2%) and depression in more than half studies (pooled prevalence rate of 22.8%); five studies reported insomnia (pooled prevalence of 38.9%). Female HCWs and nurses exhibited higher rates of anxious and depressive symptoms.

Another recent systematic review (7) highlighted that paying attention to the mental health issues, reducing the workload of HCWs by adjusting their work shifts, reducing job-related stressors, and creating a healthy work environment may prevent or reduce Burnout among HCWs that worked at 2019-nCOVID wards. Burnout is a multi-dimensional response to physiological, emotional, or interpersonal job stressors and it could lead to the development of psychological problems, increased risk of suicide, and substance use among HCWs (8). High rates of burnout have been described in HCWs working in 2019-nCOVID wards (including nurses and physicians) (9, 10), frontline HCWs, those working in emergency departments, ambulances, and intensive care units (ICUs) (11), residents in training exposed to 2019-nCOVID patients (12). Controversially, one study (13) reported significantly higher levels of burnout among physicians and nurses working in non-2019-nCOVID wards, compared to HCWs working in 2019-nCOVID wards, and, similarly, lower levels of burnout among first-line residents in training, than in those working at other wards have been described (14). A possible explanation for these findings was that first-line HCWs may perceive themselves as more valued, and feel higher levels of control over work, as they were more aware of the preventative policies and procedures.

Notwithstanding possible inconsistencies among studies, overall, the available results in the literature point to the need of addressing mental health issues in HCWs, including anxiety, mood and sleep disturbances, as well as burnout.

Many of the available studies about the current pandemic (13, 15) have been conducted in China, and it is likely that a different scenario (as far as the spread of the virus, the healthcare organization, and the socio-cultural context are concerned) might play a role in mediating the mental health impact of 2019-nCOVID on HCWs. Italy was the first European country involved in the 2019-nCOVID pandemic and one of the most severely hit regarding hospital patients' overload, hence there is an urgent need to understand the consequences of the 2019-nCOVID outbreak on Italian HCWs. Some studies have focused on the topic of HCWs mental health in Italy (16–19), but to our knowledge the literature specifically investigating HCWs burnout is still scant; some examples are detailed below (20, 21). A first account of the 2019-nCOVID psycho-physical impact on Italian HCWs, involving 330 HCWs from a health institution in Northern Italy (21) found that 26.8% of participants had clinical levels of depression, 31.3% of anxiety (even though up to 71.2% scored above the clinical cutoff for state anxiety), 34.3% of stress, 36.7% of post-traumatic stress. Burnout was measured with the Maslach Burnout Inventory (MBI), finding moderate and severe levels of emotional exhaustion (EE), depersonalization (D), and reduced personal accomplishment (PA) in 35.7% and 31.9%; 14.0% and 12.1%; 40.1% and 34.3% of participants, respectively. While all the three dimensions of burnout were predicted by work hours, psychological comorbidities, fear of infection and perceived support by friends, further predictors of emotional exhaustion and depersonalization were female gender, being a nurse, working in the hospital, contact with 2019-nCOVID patients. Moreover, younger age predicted reduced personal accomplishment.

Another study performed in a major university hospital in central Italy on 265 HCWs facing 2019-nCOVID emergency found higher levels of secondary traumatization in females, higher compassion satisfaction in frontline HCWs than in second-line ones, and an association of burnout with depression and anxiety scores (22).

One study evaluating stress levels in 102 HCWs from different rehabilitation departments (psychiatric and multidisciplinary), not specifically involved as frontline HCWs in the COVID-19 pandemic, found that higher educational level seemed to emerge as a protective factor for work (23).

A couple of Italian studies (24, 25), which were completed during the lockdown period in 2020, analyzed coping strategies, emergency stress, and secondary trauma, as well as their relationships with demographic variables and other stress factors. Nonetheless, these two studies included HCWs involved in the treatment of 2019-nCOVID patients as well as emergency workers (firefighters, civil protection, and ambulance personnel).

Our study aimed to evaluate the mental health impact of the first wave of the 2019-nCOVID pandemic on HCWs from North-Eastern Piedmont (26), an Italian high-risk area. For this purpose, we performed an online survey which was e-mailed to HCWs from different settings (hospital and community healthcare facilities, emergency and non-emergency services). The primary outcome of the study was the assessment of burnout in this sample of HCWs, while the investigation of anxiety, depression, and post-traumatic stress symptoms were secondary outcomes. According to the preliminary findings available in the literature, we expected to find high levels of burnout, anxiety, depression, and post-traumatic stress symptoms in our sample.

## Materials and Methods

The study protocol was approved by the local Ethical Board (Comitato Etico Interaziendale di Novara, Protocollo 534/CE, Studio n. CE 82/20, approved on May 11th, 2020). The primary and secondary outcomes were assessed with an online survey predisposed *ad-hoc*, including standardized and validated psychometric measures. The survey was implemented with the REDCap platform and e-mailed at the end of the first wave of the COVID pandemic emergency crisis period (in June 2020), on behalf of the human resources offices in charge of the healthcare institutions detailed below, who have access to the mailing lists including the institutional e-mail contacts of all HCWs employees. This strategy was adopted in order to offer everyone the opportunity to take part in the survey, while granting anonymity with the use of the REDCap link to fill in the survey. Data gathering closed at the end of August. We involved the following healthcare institutions of Novara, in Piedmont, in Northern Italy: Maggiore della Carità University Hospital, Community Mental Health Services, Territorial Emergency Service (118), General Practitioners (GPs) and Out of Hours Service.

The online survey presented the objectives of the research; HCWs were required to give their informed consent to participate.

The first part of the online survey included general information, questions about the professional role and possible changes in job tasks and duties during the peak of the pandemic. With more detail, information was gathered about the following: socio-demographic characteristics (gender, age, ethnicity, marital status, employment), job features (role, medical specialization for physicians, usual place/ward/outpatient service of work, collaboration with COVID services, number of working hours per week, increase in working hours compared to usual) (for more details see Supplementary Material 1). Standardized and validated self-administered measures were used for the assessment of burnout, overall health perception, distress perceived because of stressing life events, depression, and anxiety (see below for a detailed description of measures) Supplementary Material.

In our sample of HCWs, four main subgroups could be identified: medical doctors/physicians, residents in training (meaning graduated medical doctors attending specialization schools), nurses and “others” (this group included participants who did not fit any of the previous categories, such as psychologists, social workers, radiology, and laboratory technicians, educators).

### Maslach Burnout Inventory—Human Services Survey for Medical Personnel (MBI-HSS MP)

It is a 22-items scale, with each item scored on a 7-point (ranging from 0, “never,” to 6 “every day”). The MBI-HSS MP evaluates individuals' experience of occupational burnout in individuals who work with people (human services and medical professionals), with three components: emotional exhaustion (EE), depersonalization (D), and reduced personal accomplishment (PA). EE (9 items) measures feelings of being emotionally overextended and exhausted at work. D (5 items) measures an unfeeling and impersonal response toward recipients of one's service, care, treatment, or instruction. PA (8 items) measures feelings of competence and achievement in one's work with people. Burnout is suggested by high EE or D scores, and low PA ones. Each subscale score can then be coded as “low,” “average” or “high” according to the scoring key, and is considered separately from the other, without combining into a single, total score. The Cronbach's alpha was reported to be satisfactory for PA (alpha = 0.71) and EE (alpha = 0.85), and moderate for D (alpha = 0.58) (27). The Italian version was validated by Sirigatti and Stefanile (28).

### General Health Questionnaire-12 Items (29)

This is the most extensively used screening instrument for common mental disorders and a general measure of psychiatric well-being. It was developed for non-clinical populations to detect a wide range of disorders, and specifically the anxiety/depression spectrum; it is a valid and reliable instrument across cultures. The Italian version was validated by Fraccaroli et al. (30). GHQ-12 items are rated on a 4-point scale (“less than usual,” “no more than usual,” “rather more than usual,” “much more than usual”) offering a total score ranging from 0 to 36 points, with higher scores indicating worse mental well-being (31). The GHQ-12 Cronbach's alpha is 0.9. Factor analysis reveals two significant components which accounted for 59% of the variance (32).

### Impact of Event Scale (IES) (33)

It consists of 15-items, rated on a 4-point scale according to how often each has occurred in the past 7 days (0 = not at all; 1 = rarely; 3 = sometimes; 5 = often). All IES items anchored to a specific stressor (34). Besides the IES total subjective stress score, two subscales are identified, one for intrusive symptoms (intrusive thoughts, nightmares, intrusive feelings, and imagery; seven items, scores ranging from 0 to 35), and one for avoidance symptoms (numbing of responsiveness, avoidance of feelings, situations, ideas; eight items, scores ranging from 0 to 40). The IES can be used for repeated measurement, showing a sensitivity to change which makes it useful to assess progress in therapy (35). A cutoff point of 26 is suggested, with a moderate or severe impact suggested by scores above the cutoff. Both the intrusion and avoidance scales have displayed acceptable reliability (alpha of 0.79 and 0.82, respectively), and split-half reliability for the whole scale of 0.86 (33). The IES has also displayed the ability to discriminate a variety of traumatized groups from non-traumatized groups (28). The Italian version was validated by Pietrantonio et al. (36). The questionnaire evaluating trauma used in this study was not the Impact of Event Scale with modifications for 2019-nCOVID (IES-COVID19) (37), but in the protocol was specified to answer to the IES questions considering “event” how everything that was related to the 2019-nCOVID pandemic.

### Beck Anxiety Inventory (BAI) (38)

The BAI is a 21-item scale for the assessment of anxiety symptoms severity. Each item is rated on a 4-point scale (from 0 = not at all to 3 = severely, can barely stand it), focusing on the past week experience. The final score is obtained from the sum of the individual items and is between 0 and 63 (≤21 = minimum level of anxiety; 22–35 = medium level of anxiety; ≥36 = high level of anxiety). The BAI test showed high internal consistency (Cronbach α = 0.94) and is acceptably reliable over an average time lapse of 11 days (*r* = 0.67) consistency (39). The Italian version was validated by Sica and Ghisi (40).

### Beck Depression Inventory (BDI-II) (41)

The BDI is a 21-item self-report inventory measuring the severity of depression. Each item is scored on a 4-point scale, as for the BAI. The higher the total score, the more severe is depression; standardized cutoff values are the following: 0–13 = minimal depression; 14–19 = mild depression; 20–28= moderate depression; 29–63= severe depression. The BDI-II, with an overall Cronbach's alpha of 0.90 and composite reliability of 0.91, has an adequate internal consistency (42). The Italian version was validated by Sica and Ghisi (40).

### Statistical Analyses

Continuous data were synthesized as median (I, III quartiles); categorical data were summarized as percentages and absolute frequencies. The Wilcoxon-type tests were performed for continuous variables and the Pearson chi-square test, or Fisher exact test, as appropriate, for categorical ones. The categorized scores were considered as endpoints. A Proportional Odds Model was estimated for the ordinal responses with more than two categories. The model estimated univariable Odds Ratios (OR) together with the 95% confidence interval, and the *p*-values were reported; the multivariable estimated OR have been also computed. The continuous scores were modeled with univariable and multivariable Ordinary Least Square (OLS) methods reporting the estimates with the relative 95% confidence intervals. The relevant predictors in the multivariable models were selected via the backward elimination method. The possible inflation of the type I error rate has been controlled by performing a Benjamini Hochberg correction for multiple outcomes for both univariable and multivariable models. The computations were performed using the software R 4.0.2 [1] with the rms [2] package (43, 44).

## Results

The online survey was e-mailed to 2,422 HCWs and filled in by 897 (37%) of them. Only 653 out of these 897 (73%) properly completed all the questionnaires. The 244 incomplete records were thus excluded from the statistical analyses. Statistically significant results of the analyses performed are reported in **Tables 2**–**7**.

Most participants were aged 30–49 years (51%), female (68%), married/with partner (63%), with children (55%) (Table 1). As far as employment is concerned, the higher percentage of respondents was that of nurses (21%). Most respondents had a 2019-nCOVID positive nasal swab (86%) and in the 81% of cases they reported 2019-nCOVID-related symptoms. For more details see Table 1.

**Table 1:** Descriptive data of the sample, including socio-demographic and work related variables.

**Variables**		***N***	**%**
Gender	Male	210	32.2%
	Female	443	67.8%
Age categories	18–29 years	92	14.1%
	30–39 yeasr	189	28.9%
	40–49 years	145	22.2%
	≥50 years	227	34.8%
Marital status	Single/divorced/widow	159	24.3%
	Married/cohabitant	413	63.2%
	In a stable relationship	81	12.4%
Chlidren	Yes	358	54.8%
	No	295	45.2%
Working categories	Doctor	286	43.8%
	Residents in training	99	15.2%
	Nurse	137	21.0%
	Other^*^	131	20.1%
Positivity to COVID-19 swab	Yes	564	86.4%
	No	89	13.6%
COVID-19 related symptoms	Yes	528	80.9%
	No	125	19.1%
Health problems not related to COVID-19	Yes	556	85.1%
	No	97	14.9%
Changing of mansion due to the COVID-19 pandemic	Yes	89	13.6%
	No	564	86.4%
Job modification due to the COVID-19 pandemic	Yes	322	49.3%
	No	331	50.7%
Someone of dear positive to COVID-19 buffer	Yes	454	69.5%
	No	199	30.5%
Modification of family habits for fear of infecting loved one dear	Yes	43	6.6%
	No	525	80.4%
	No answer	85	13.0%

*N, number of patients*.

*%, percentage of individuals*.

* *, psychologists, socio-health, psychological, radiological and laboratory technicians, educators*.

Scores suggesting the presence of burnout were found in all the three subscales of the MBI. With more detail, the median value for the EE scale was 20.0 (moderate level of burnout), the median value for D scale was 10.9 (moderate level of burnout), the median value for PA scale was 31.8 (high level of burnout). Moderate-high levels were self-reported by 51 and 86% of HCWs for EE and D, respectively, while 94% of the sample had moderate-low levels of PA (Tables 1, 2).

**Table 2:** Focus on burnout as measured with the Maslach Burnout Inventory (MBI) scale: analysis of categorical socio-demographic features and test results (*n*, % column).

			**BURNOUT**											
***N*** **=** **653**			**MBI—EE**				**MBI—D**				**MBI—PA**			
**Cut-off**, ***N***			**Low**	**Medium**	**High**	***p***	**Low**	**Medium**	**High**	***p***	**Low**	**Medium**	**High**	***p***
		***N =* 653 (100%)**	***N =* 318**	***N =* 207**	***N =* 128**		***N =* 91**	***N =* 289**	***N =* 273**		***N =* 38**	***N =* 182**	***N =* 433**	
Gender	Males	210 (32%)	120 (38%)	54 (26%)	36 (28%)	0.011								
	Females	443 (68%)	198 (62%)	153 (74%)	92 (72%)									
Cat HW	Medical Doctors/Physicianss	286 (44%)					31 (34%)	136 (47%)	119 (44%)	0.002				
	Residents in trainings	99 (15%)					8 (9%)	41 (14%)	50 (18%)					
	Nurses	137 (21%)					33 (36%)	60 (21%)	44 (16%)					
	Other	131 (20%)					19 (21%)	52 (18%)	60 (22%)					
BAI	Minimum	579 (89%)	312 (98%)	180 (87%)	87 (68%)	<0.001	90 (99%)	276 (96%)	213 (78%)	<0.001				
	Medium	58 (9%)	6 (2%)	25 (12%)	31 (24%)		1 (1%)	13 (4%)	48 (18%)					
	High	12 (2%)	0 (0%)	2 (1%)	10 (8%)		0 (0%)	0 (0%)	12 (4%)					
BDI	Minimal	471 (72%)	285 (90%)	133 (64%)	53 (41%)	<0.001	80 (88%)	231 (80%)	160 (59%)	<0.001	30 (79%)	149 (82%)	292 (67%)	0.02
	Low	104 (16%)	25 (8%)	48 (23%)	31 (24%)		8 (9%)	38 (13%)	58 (21%)		4 (11%)	19 (10%)	81 (19%)	
	Moderate	51 (8%)	7 (2%)	20 (10%)	24 (19%)		3 (3%)	17 (6%)	31 (11%)		3 (8%)	10 (5%)	38 (9%)	
	High	27 (4%)	1 (0%)	6 (3%)	20 (16%)		0 (0%)	3 (1%)	24 (9%)		1 (3%)	4 (2%)	22 (5%)	
IES	Subclinical	207 (32%)	139 (44%)	45 (22%)	23 (18%)	<0.001	39 (43%)	99 (34%)	69 (25%)	<0.001				
	Mild	213 (32%)	104 (33%)	66 (32%)	43 (34%)		29 (32%)	96 (33%)	88 (32%)					
	Moderate	148(23%)	50 (16%)	66 (32%)	32 (25%)		14 (15%)	66 (23%)	68 (25%)					
	Severe	85 (13%)	25 (8%)	30 (14%)	30 (23%)		9 (10%)	28 (10%)	48 (18%)					
GHQ	No problem	53 (8%)	42 (13%)	8 (4%)	3 (2%)	<0.001	10 (11%)	31 (11%)	12 (4%)	<0.001	3 (8%)	20 (11%)	30 (7%)	0.01
	Some problems	330 (51%)	196 (62%)	102 (49%)	32 (25%)		55 (60%)	158 (55%)	117 (43%)		22 (58%)	105 (58%)	203 (47%)	
	Several problems	270 (41%)	80 (25%)	97 (47%)	93 (73%)		26 (29%)	100 (35%)	144 (53%)		13 (34%)	57 (31%)	200 (46%)	

*Only statistically significant differences (p <0.05) are shown*.

*HCWS, Healthcare Worker; MBI, Maslach Burnout Inventory; EE, Emotional Exhaustion; D, Depersonalization; PA, Personal Accomplishment; BAI, Beck Anxiety Inventory; BDI, Beck Depression Inventory; IES, Impact of Event Scale; GHQ, General Health Questionnaire*.

*Cut-off scores*

*MBI—EE: Low ≤ 17, Med 18–29, High ≥30*.

*MBI—D: Low ≤ 05, Med 6–11, High ≥12*.

*MBI—PA: Low ≥40, Med 35–39, High ≤ 34*.

*BECK ANXIETY INVENTORY Minimum 0–21, Medium 22–35, High ≥ 36*.

*BECK DEPRESSION INVENTORY Minimal 0–13, Mild 14–19, Moderate 20–28, Severe 29–63*.

*IMPACT OF EVENT SCALE Subclinical 0–8, Mild 9–25, Moderate 26-43, Severe ≥ 44*.

*GENERAL HEALTH QUESTIONNAIRE No problem 0–14, Some problems 15–19, Several problems 20–36*.

Higher levels of burnout were found in females, in those aged <30 years, in HCWs exposed to changes in their daily and family habits, in those who had to change their duties at work and in residents in trainings. With more detail, females showed higher EE than males (as suggested by both the continuous and categorical analyses of this variable: mean EE scores higher in females; higher rate of females with medium-high EE levels, 55 vs. 43%). Furthermore, female gender was a risk factor for high EE (OR males vs. females = 0.645, IC95%:0.47–0.89, *p* = 0.01). Higher levels of D were found in the age grou*p* < 30 years than in the other ones (30–49 years and ≥50 years) (*p* = 0.04) (OR 18–29 vs. 30–49 = 1.51, IC95% 0.97–2.37, *p* = 0.05) and in those HCWs whose family habits changed during the pandemic (*p* = 0.032) (Tables 3, **6**).

**Table 3:** Wilcoxon type comparison of median test scores in the sample (N = 653), according to the socio-demographic and COVID-19-related information.

***N*** **=** **653**		**MBI**						**BAI**	***p***	**BDI**	***p***	**IES**	***p***	**GHQ**	***p***
		**EE**	***p***	**D**	***p***	**PA**	***P***								
Age	>50 years			10.00	0.04			7.00	0.02						
	30–49 years			10.50				8.00							
	18–29 years			12.00				9.50							
Gender	Males	16.00	<0.001					5.00	<0.001	5.00	<0.001	9.00	<0.001	18.00	<0.001
	Females	19.00						9.00		9.00		23.00		19.00	
Marital Status	Married or cohabiting							9.00	0.05						
	Lives alone							7.00							
Children	No			11.00	0.01										
	Yes			10.00											
HCWS Category	Medical Doctors/Physicianss			10.00	<0.001	33.00	0.014	7.00	0.026			17.00	0.011		
	Residents in trainings			12.00		31.00		9.00				15.00			
	Nurses			8.00		32.00		8.00				24.00			
	Other			11.00		33.00		8.00				20.00			
Changes in Job	No	17.00	0.01	9.00	0.01					7.00	0.05	16.00	0.02		
	Yes	19.00		11.00						8.00		20.00			
Changes in Family Habits	No			9.00	0.032			4.00	<0.001	4.00	<0.001	9.00	<0.001	17.00	<0.001
	Yes			11.00				8.00		9.00		20.00		19.00	
Positivity 2019-nCOV	No														
	Yes														
COVID-19 symptoms	No														
	Yes														
Non-COVID health problems	No							7.00	<0.001	7.00	<0.001	18.00	0.005	18.00	<0.001
	Yes							13.00		13.00		23.00		20.00	
Family members positive to 2019-nCOV	No							7.00	0.0059	7.00	0.0046	17.00	0.005	18.00	0.005
	Yes							9.00		10.00		22.00		19.00	

*Only statistically significant differences (p <0.05) are shown*.

*HCWS, Healthcare Worker; MBI, Maslach Burnout Inventory; EE, Emotional Exhaustion; D, Depersonalization; PA, Personal Accomplishment; BAI, Beck Anxiety Inventory; BDI, Beck Depression Inventory; IES, Impact of Event Scale; GHQ, General Health Questionnaire*.

HCWs who had to adapt to different job duties during the pandemic emergency, compared to those who did not change their working habits, showed significantly higher scores on both the EE and D scales (EE *p* = 0.01; D *p* = 0.01); the same result was found in residents in training (D *p* < 0.001; PA *p* = 0.014), 92% of whom had medium-high scores on the MBI D subscale compared to the other professional groups (medical doctors/physicians = 89%; other = 85%; nurses 76%; *p* = 0.002). This last result was supported also by the univariate analysis (D – OR residents in training vs. medical doctors/physicians = 1.40, IC95% 0.90–2.16, *p* = 0) (Tables 3, **6**).

When analyzing subgroups of HCWs, the following results emerged. Residents in trainings showed on average significantly higher anxiety scores (BAI) than the other categories (*p* = 0.026), although the percentage (11%) showing moderate/high levels on BAI is lower than in other categories, except for physicians (moderate/high BAI = 18% in other; 15% in nurses; 7% in physicians; *p* = 0.022). Levels of distress were higher in nurses (*p* = 0.011). As regards the context outside the workplace, married/cohabiting HCWs, compared to those who lived alone, showed higher levels of anxiety (*p* = 0.05). A particular risk factor for adverse clinical outcomes appears to be changes in family or non-work habits. In fact, HCWs who changed their off-work habits showed higher levels of anxiety, depressive, and stress-related symptoms and overall worse mental health (*p* < 0.001). These data are also confirmed by the analysis of categorical data: in 93% of the HCWs who changed the job, a GHQ score indicative of moderate/severe problems was observed (83% in those who did not change their job, *p* < 0.001) and in 38% distress symptoms (19% in those who have not changed their work habits, *p* = 0.028) (Tables 3, 4).

**Table 4:** Focus on Anxiety, Depression, Stress, and Mental Health: analysis of categorical socio-demographic features (*n*, % column).

**Variable**			**BAI**				**BDI**					**IES**					**GHQ**			
			**Minimum**	**Medium**	**High**	***p***	**Minimal**	**Mild**	**Moderate**	**Severe**	***P***	**Subclinical**	**Mild**	**Moderate**	**Severe**	***p***	**No problem**	**Some problems**	**Several problems**	***p***
		***N* = 653 (100%)**	***N =* 318**	***N =* 207**	***N =* 128**		***N =* 471**	***N =* 104**	***N =* 51**	***N =* 27**		***N =* 207**	***N =* 213**	***N =* 148**	***N =* 85**		***N =* 53**	***N =* 330**	***N =* 270**	
Gender	Males	220 (32%)	198 (34%)	8 (13%)	4 (33%)	0.003	173 (37%)	22 (21%)	8 (16%)	7 (26%)	<0.001						26 (49%)	120 (36%)	64 (24%)	<0.001
	Females	445 (68%)	381 (66%)	54 (87%)	8 (67%)		298 (63%)	82 (79%)	43 (84%)	20 (74%)							27 (51%)	210 (64%)	206 (76%)	
Cat. HCWS	Medical Doctors/Physicianss	286 (44%)	267 (46%)	16 (26%)	3 (25%)	0.022														
	Resi-dents	99 (15%)	88 (15%)	8 (13%)	3 (25%)															
	Nurses	137 (21%)	116 (20%	18 (29%)	3 (25%)															
	Other	131 (20%)	108 (19%)	20 (32%)	3 (25%)															
Changes in Habits	No	89 (14%)										41 (20%)	31 (15%)	13 (9%)	4 (5%)	0.028	15 (28%)	57 (17%)	17 (6%)	<0.001
	Yes	564 (86%)										166 (80%)	182 (85%)	135 (91%)	81 (95%)		38 (72%)	273 (83%)	253 (94%)	
Non-COVID health problems	No	556 (85%)					419 (89%)	74 (71%)	41 (80%)	22 (81%)	<0.001	188 (91%)	176 (83%)	125 (84%)	67 (79%)	0.001	48 (91%)	290 (88%)	218 (81%)	0.026
	Yes	97 (15%)					52 (11%)	30 (29%)	10 (20%)	5 (19%)		34 (16%)	37 (17%)	38 (26%)	16 (19%)		5 (9%)	40 (12%)	52 (19%)	
Family mem-bers positive to 2019-nCOV	No	454 (70%)															42 (79%)	238 (72%)	174 (64%)	0.035
	Yes	199 (30%)															11 (21%)	92 (28%)	96 (36%)	

*Only statistically significant differences (p <0.05) are shown*.

*Cat. HCWS, Category Healthcare Worker; BAI, Beck Anxiety Inventory; BDI, Beck Depression Inventory; IES, Impact of Event Scale; GHQ, General Health Questionnaire; p, p-Value; Min, Minimal; Mod, Moderate*.

*Cut-off scores*

*BECK ANXIETY INVENTORY Minimum 0–21, Medium 22–35, High ≥ 36*.

*BECK DEPRESSION INVENTORY Minimal 0–13, Mild 14–19, Moderate 20–28, Severe 29–63*.

*IMPACT OF EVENT SCALE Subclinical 0–8, Mild 9–25, Moderate 26–43, Severe ≥ 44*.

*GENERAL HEALTH QUESTIONNAIRE No problem 0–14, Some problems 15–19, Several problems 20–36*.

Having general health problems, not related to 2019-nCOVID infection, as well as the presence of family members who have contracted the 2019-nCOVID, played a significant role on mental health outcomes; in both cases, in fact, significantly higher scores were observed in all scales (in general *p* < 0.05). If we analyze the categorical data, among the HCWs who had non-COVID health problems, 15% showed depressive symptoms (11% in those who did not have such problems, *p* < 0.001), 43% stress symptoms (34 % of those who did not have health problems). Ninety-four percentage of HCWs having family members who have contracted the 2019-nCOVID disease reported poor mental health (vs. 91% of those who have not had infected family members) (Tables 3, 4).

A poor mental health status, post-traumatic symptoms, anxiety, and depression were perceived by 92, 36, 10, and 10% of HCWs, as suggested by the GHQ (median GHQ = 19.4), IES (median IES = 21.0), BAI (median BAI = 10.3), and BDI (median BDI = 9.8) scores, respectively (Table 5).

**Table 5:** Focus on Anxiety, Depression, Stress, and Mental Health: analysis of categorical tests (IES, GHQ) data (*n*, % column).

**Variable**			**BAI**			**BDI**				**IES**				**GHQ**		
			**Minimum**	**Medium**	**High**	**Minimal**	**Mild**	**Moderate**	**Severe**	**Subclinical**	**Mild**	**Moderate**	**Severe**	**No problem**	**Some problems**	**Several problems**
		**N (%) 653**	***N =* 318**	***N =* 207**	***N =* 128**	***N =* 471**	***N =* 104**	***N =* 51**	***N =* 27**	***N =* 207**	***N =* 213**	***N =* 148**	***N =* 85**	***N =* 53**	***N =* 330**	***N =* 270**
BAI	Low (0–21)	579 (89%)				462 (98%)	84 (81%)	27 (53%)	6 (22%)	206 (100%)	200 (94%)	122 (82%)	51 (60%)	52 (98%)	316 (96%)	211 (78%)
	Moderate (22–35)	62 (9%)				9 (2%)	20 (19%)	21 (41%)	12 (44%)	1 (0%)	13 (6%)	25 (17%)	23 (27%)	1 (2%)	12 (4%)	49 (18%)
	High (>36)	12 (2%)				0 (0%)	0 (0%)	3 (6%)	9 (33%)	0 (0%)	0 (0%)	1 (1%)	11 (13%)	0 (0%)	2 (1%)	10 (4%)
BDI	Minimal (0–13)	479 (73%)								191 (41%)	166 (35%)	86 (18%)	28 (6%)	49 (92%)	297 (90%)	125 (46%)
	Low (14–19)	104 (16%)								15 (14%)	33 (32%)	38 (37%)	18 (17%)	3 (6%)	26 (8%)	75(28%)
	Moderate (20–28)	51 (7%)								1 (2%)	11 (22%)	17 (33%)	22 (43%)	1 (2%)	5 (2%)	45 (17%)
	High (29–63)	27 (4%)								0 (0%)	3 (11%)	7 (26%)	17 (63%)	0 (0%)	2 (1%)	25 (9%)
IES	Minimal (0–8)	207 (32%)												24 (45%)	140 (42%)	43 (16%)
	Low (9–25)	213 (32%)												17 (32%)	122 (37%)	74 (27%)
	Moderate (26–43)	148 (23%)												9 (17%)	48 (15%)	91 (34%)
	High (>44)	85 (13%)												3 (6%)	20 (6%)	62 (23%)
GHQ	Low (0–14)															
	Moderate (15–19)															
	High (20–36)															

*Only statistically significant differences (p <0.05) are shown; for all comparisons a p <0.001 was observed*.

*BAI, Beck Anxiety Inventory; BDI, Beck Depression Inventory; IES, Impact of Event Scale; GHQ, General Health Questionnaire; p, p-Value; Min, Minimal; Mod, Moderate*.

*Cut-off scores*

*BECK ANXIETY INVENTORY Low 0–21, Moderate 22–35, High ≥ 36*.

*BECK DEPRESSION INVENTORY Minimal 0–13, Mild 14–19, Moderate 20–28, Severe 29–63*.

*IMPACT OF EVENT SCALE Subclinical 0–8, Mild 9–25, Moderate 26–43, Severe ≥ 44*.

*GENERAL HEALTH QUESTIONNAIRE No problem 0–14, Some problems 15–19, Several problems 20–36*.

The univariate analysis identified male gender as a protective factor for adverse mental health outcomes (males vs. females: BAI OR = 0.38, IC95% = 0.20–0.72, *p* < 0.01; BDI OR = 0.44, IC95% = 0.30–0.67, *p* < 0.01; IES OR = 0.33, IC95% = 0.24–0.45, *p* < 0.01; GHQ OR = 0.49, IC95% = 0.36–0.68, *p* < 0.01). More severe stress was found in HCWs who had to change their duties at work during the pandemic emergency, and in those who experienced changes also in their daily and family habits. The univariate analysis supported the protective role of not undergoing changes in usual habits (no change vs. change: BAI OR = 0.33, IC95% = 0.12–0.92, *p* = 0.04; BDI OR = 0.35, IC95% = 1.19–0.67, *p* < 0.01; IES OR = 0.44, IC95% = 0.29–0.67, *p* = 0; GHQ OR = 0.31, IC95% = 0.20–0.50, *p* < 0.01) (Table 6).

**Table 6:** Results of the univariate analysis.

			**OR**	**IC (95%)**	**χ2**	**Df**	***p***
**MBI EE**	Gender	Males vs. Females	0.64753	0.47133–0.8896	7.19	1	0.01
	Changes in Habits	No vs. Yes	0.60242	0.39005–0.9304	5.22	1	0.02
**MBI D**	Age	18–29 vs. 30–49	1.5157	0.97077–2.3664	5.95	2	0.05
		>50 vs. 30–49	0.84805	0.61678–1.166			
	Children	No vs. Yes	1.3791	1.0291–1.8481	4.63	1	0.03
	Category HCWSS	Residents in trainings s vs. Medical Doctors/Physicians	1.399	0.90478–2.1631	15.17	3	<0.001
		Nurses vs. Medical Doctors/Physicians	0.55424	0.37501–0.81914			
		Other vs. Medical Doctors/Physicians	1.0595	0.71372–1.5729			
**BAI**	Gender	Males vs. Females	0.37872	0.19941–0.719–26	8.8	1	<0.001
	Non-COVID health problems	Yes vs. No	2.0312	1.137-3.6288	5.73	1	0.02
	Changes in Habits	No vs. Yes	0.32935	0.1172–0.92551	4.44	1	0.04
	Category HCWSS	Residents in trainings vs. Medical Doctors/Physicians	1.7812	0.81609–3.8877	12.57	3	0.01
		Nurses vs. Medical Doctors/Physicians	2.5327	1.313–4.8855			
		Other vs. Medical Doctors/Physicians	2.9672	1.5546–5.6635			
**BDI**	Gender	Males vs. Females	0.44514	0.29718–0.66675	15.43	1	<0.001
	Non-COVID health problems	Yes vs. No	2.299	1.5117–3.4962	15.15	1	<0.001
	Family members positive to 2019-nCOV	Yes vs. No	1.6436	1.1549–2.3392	7.62	1	0.01
	Changes in Habits	No vs. Yes	0.35532	0.18893–0.66826	10.31	1	<0.001
**IES**	Gender	Males vs. Females	0.32912	0.24116–0.44916	49.07	1	<0.001
	Non-COVID health problems	Yes vs. No	1.6473	1.1222–2.4182	6.5	1	0.01
	Family members positive to 2019-nCOV	Yes vs. No	1.4553	1.0775–1.9655	5.99	1	0.01
	Changes in habits	No vs. Yes	0.44286	0.29331–0.66865	15.01	1	<0.001
	Category HCWSS	Residents in trainings s vs. Medical Doctors/Physicians	0.90816	0.59565–1.3846	9.23	3	0.03
		Nurses vs. Medical Doctors/Physicians	1.6666	1.157–2.4006			
		Other vs. Medical Doctors/Physicians	1.0876	0.74868–1.5801			
**GHQ**	Gender	Males vs. Females	0.49433	0.35698–0.68452	18	1	<0.001
	Non-COVID health problems	Yes vs. No	1.3184	0.90194–1.9273	2.04	1	0.01
	Family members positive to 2019-nCOV	Yes vs. No	1.5287	1.1053–2.1143	6.58	1	0.01
	Changes in Habits	No vs. Yes	0.31512	0.20017–0.49608	24.88	1	<0.001

*MBI, Maslach Burnout Inventory; EE, Emotional Exhaustion; D, Depersonalization; GHQ, General Health Questionnaire; BDI, Beck Depression Inventory; BAI, Beck Anxiety Inventory; IES-R, Impact of Event Scale Revised; HCW, health workers; OR, Odds Ratio; CI, Confidence Interval; p, p-value*.

*Cut-off scores*

*MBI—EE: Low ≤ 17, Med 18–29, High ≥30*.

*MBI—D: Low ≤ 05, Med 6–11, High ≥12*.

*MBI—PA: Low ≥40, Med 35–39, High ≤ 34*.

*BECK ANXIETY INVENTORY Low 0–21, Moderate 22–35, High ≥ 36*.

*BECK DEPRESSION INVENTORY Minimal 0–13, Mild 14–19, Moderate 20–28, Severe 29–63*.

*IMPACT OF EVENT SCALE Subclinical 0–8, Mild 9–25, Moderate 26–43, Severe ≥ 44*.

*GENERAL HEALTH QUESTIONNAIRE No problem 0–14, Some problems 15–19, Several problems 20–36*.

The multivariate analysis substantially confirmed the results from the univariate analysis identifying male gender as a protective factor for adverse mental health outcomes (males vs. females: BAI OR = 0.38, IC95% = 0.30–0.72, *p* < 0.01; BDI OR = 0.45, IC95% = 0.30–0.67, *p* < 0.01; IES OR = 0.35, IC95% = 0.26–0.49, *p* < 0.01; GHQ OR = 0.51, IC95% = 0.37–0.71, *p* = 0.01, MBI EE OR:0.65, IC 95% = 0.47–0.89, *p* = 0.01). More severe stress and burnout (D) were found in medical doctors/physicians than other HCWs categories (GHQ *p* = 0.020; MBI D *p* < 0.01). More severe depression, mental health problems and distress were found in HCWs who had health problems not related to 2019-nCOVID (BDI OR = 2–29, IC95% = 1.50–3.50, *p* < 0.001; GHQ OR = 2.02, IC95% = 1.3–3.15, *p* < 0.001; IES OR = 1.58, IC95% = 1.07–2.33, *p* = 0.02). Finally, HCWs who changed their family habits due to COVID 19 showed more severe mental health problems (GHQ OR = 0.35, IC95% = 0.22–0.55, *p* < 0.001), as did HCWs who changed their family habits due to the fear of infecting loved ones, who showed higher distress levels (IES OR = 0.54, IC95% = 0.30–0.98, p = 0.01) (Table 7).

**Table 7:** Results of the multivariate analysis.

			**OR**	**IC (95%)**	**χ2**	**Df**	***p***
**MBI D**	Categories HCWs/physicians	Residents in training vs. medical doctors/physicians	1.399	(0.90478–2.1631)	15.17	3	<0.001
		Nurses vs. medical doctors/physicians	0.55424	(0.37501–0.81914)			
		Others vs. medical doctors/physicians	1.0595	(0.71372–1.5729)			
**MBI EE**	Gender	Male vs. female	0.64753	(0.47133–0.8896)	7.19	1	0.01
**BAI**	Gender	Male vs. female	0.37872	(0.19941–0.71926)	8.8	1	<0.001
**BDI**	Gender	Male vs. female	0.44647	(0.29719–0.67071)	15.08	1	<0.001
	Health problems not related to COVID−19	Yes vs. no	2.2887	(1.4989–3.4948)	14.7	1	<0.001
**IES**	Gender	Male vs. female	0.35362	(0.25809–0.48452)	41.86	1	0
	Health problems not related to COVID-19	Yes vs. no	1.581	(1.0696–2.3369)	5.28	1	0.02
	Family members positive to 2019-nCOV	Yes vs. no	1.4084	(1.0383–1.9103)	4.85	1	0.03
	Changing of family habits for fear of infecting loved ones	No vs. yes	0.54081	(0.29925–0.97734)	10.38	2	0.01
		No answer vs. yes	0.55438	(0.36118–0.85092)			
**GHQ**	Gender	Male vs. female	0.51319	(0.36652–0.71857)	15.09	1	<0.001
	Health problems not related to COVID-19	Yes vs. no	2.0234	(1.3–3.1494)	9.75	1	<0.001
	Change of family habits due to COVID−19 pandemic	No vs. Yes	0.34966	(0.22098–0.55329)	20.14	1	<0.001
	Categories HCWs	Residents in training vs. medical doctors/physicians	0.15378	(-0.307961–0.61553)	10.39	3	0.020
		Nurses vs. medical doctors/physicians	0.59158	(0.39181–0.8932)			
		Others vs. medical doctors/physicians	0.66587	(0.44323–1.0003)			

*MBI, Maslach Burnout Inventory; EE, Emotional Exhaustion; D, Depersonalization; GHQ, General Health Questionnaire; BDI, Beck Depression Inventory; BAI, Beck Anxiety Inventory; IES-R, Impact of Event Scale Revised; HCW, health workers; OR, Odds Ratio; CI, Confidence Interval; p, p-value*.

*Cut-off scores*

*MBI–EE: Low ≤ 17, Med 18–29, High ≥30*.

*MBI–D: Low ≤ 05, Med 6–11, High ≥12*.

*MBI–PA: Low ≥40, Med 35–39, High ≤ 34*.

*BECK ANXIETY INVENTORY Low 0–21, Moderate 22–35, High ≥ 36*.

*BECK DEPRESSION INVENTORY Minimal 0–13, Mild 14–19, Moderate 20–28, Severe 29–63*.

*IMPACT OF EVENT SCALE Subclinical 0–8, Mild 9–25, Moderate 26–43, Severe ≥ 44*.

*GENERAL HEALTH QUESTIONNAIRE No problem 0–14, Some problems 15–19, Several problems 20–36*.

## Discussion

The response rate for this survey was a little bit lower than that reported by similar studies performed in other countries (45–47). Regarding respondents' socio-demographic characteristics, our data were consistent with previous findings, concerning age, gender, marital status, type of employment and change of work, and family habits (15, 45, 48–51). More specifically, in the current study, we found that females HCWs, those aged <30 years, who was exposed to changes in duties at work had not only higher risk of burnout, but also more severe anxiety, depressive, and post-traumatic stress symptoms; furthermore, they had an overall worse self-rated general health.

The current literature worldwide suggests a high variability of results, even though there is consistent evidence about the adverse psychological outcomes of the 2019-nCOVID pandemic (9, 52–54). It is also likely that part of this variability depends on different research methods, different populations involved and different tools for the assessment of psychological outcomes.

### Burnout

Most of the studies available in the existing literature about the pandemic-related burnout used the MBI or an MBI elaboration (e.g., the MBI- Medical Personnel) (9, 13, 14, 55, 56), as in the current study. Nonetheless, the samples recruited in these studies were highly heterogenous regarding the types of HCWs involved, thus hindering the possibility to compare results.

Previous research in “ordinary” periods reported much lower rates of EE, D and PA (32, 53, 32%, respectively) in a sample of general practitioners (57), than the ones we found in the current study. Furthermore, the Italian study by Barello and coworkers (21) involving Italian healthcare workforce who directly assisted 2019-nCOVID infected patients, found higher levels of EE compared to the normative values. The results concerning female gender and younger age as risk factors for higher levels of burnout are consistent with those reported also by other researchers who found that residents in training experienced higher levels of burnout than other groups of HCWs, just as we did (9, 14, 55, 56).

Overall, Hu et al. (9), Azoulay et al. (55), and Luceño-Moreno et al. (56) found slightly higher rates of high EE in their samples (53, 64, 60%, respectively), lower D rates (57, 35, 42, respectively) and lower PA rates (67, 91.6, 60.5%, respectively) compared to our results (EE 51 %, D 86%; PA 94%). While differences in EE are slight and our scores are substantially overlapping to those found in the literature (33, 38, 41), the very high levels of burnout we found for D and PA are an interesting finding. Some hypotheses can be proposed. First, the composition of the sample deserves to be considered: our sample included a high percentage of females, who are acknowledged to be more exposed to the risk of burnout. A high rate of HCWs (more than half) had to change their job duties during the pandemic emergency furthermore, 15% of our sample was composed by residents in training. We can hypothesize that in the very critical situation they faced in delivering the appropriate care to patients both in the hospital and in healthcare facilities outside the hospital, HCWs in our sample reacted by distancing themselves from patients in order to go on with their job duties, but at the same time, while doing so, they also felt less accomplishment in their profession. We cannot exclude that cultural issues as well as the period when the survey was proposed (after the first peak of the pandemic) played a role on the results we found. The psychological distress response of HCWs to the infectious diseases may include feelings of vulnerability or loss of control and concerns about health of self, spread of virus, health of family and others, changes in work, and being isolated. Also, the social-media pressure is significative and impacts on frontline and second-line HCWs. With more detail, frontline HCWs, already used to working in emergency settings and in critical situations, proved less vulnerable to burnout during the current pandemic (13, 14, 55). Even though exposed to an increased workload, it is likely that feeling to have the situation “under control” might play a protective role for frontline HCWs (27), who may also experience a greater professional accomplishment, since they have the opportunity to apply their knowledge in their daily clinical practice and obtaining acknowledgment for that (58). On the other hand, those HCWs who continued to work in their usual job context and those who were compelled to change their job duties seemed to be more exposed and vulnerable to stressful situations. Those who remained in their usual setting likely experienced limitations in treating their patients as usual, considering that several human and practical resources were re-located according to the needs emerging from the pandemic situation. Those who changed their job duties, often found themselves involved in unfamiliar tasks, at increased risk of making mistakes, thus leading to stress and reduced professional accomplishment.

### Anxiety, Depression, Distress

The currently available literature has reported higher rates of anxiety and depression than the ones we found in our HCWs sample (about 10% of high scorers on the BAI and BDI). Other researchers have reported higher rates of depressive, anxious and stress-related symptoms [e.g., 27.2, 18.6, 24.7% (12), and 5.3, 20.7, 83% (56); 30.2 and 46.5% for depression and anxiety (55)]. The results presented by Hu and coworkers (9) were more similar to the ones we found, with moderate-severe anxiety and depression reported by 14 and 11% of the HCWs, respectively, using the Self-Rating Anxiety Scale (SAS) and Self-Rating Depression Scale (SDS). Furthermore, a recent systematic review reported the following lowest-highest prevalence values of anxiety, depression, and stress among HCWs: 24.1–67.55, 12.1–55.89, and 29.8–62.99%, respectively (59).

First of all, we cannot underestimate the fact that different tools have been used for the assessment of anxiety and depression; moreover, not all the studies have assessed stress symptoms and overall health. Besides different research methodology, the heterogeneity in the composition of the study samples is another relevant issue.

In a recent review, Carmassi et al. (60) analyzed the Post- Traumatic Stress Disorder (PTSD and Post-traumatic Stress Symptoms (PTSS) in HCWs, during the current 2019-nCOVID pandemic, as well as during two other major recent Coronavirus outbreaks (the SARS and the MERS). The review showed a higher risk for PTSD among HCWs during the current pandemic. Among the risk factors for PTSD and PTSS symptoms, the review highlighted the following: unpredictability of daily caseloads, management of patients and their families' expectations in unexpected critical cases/situations, decision-making burden, high daily fatality rates, constant changes, and updates of hospital procedures. Another review (16) reported trauma-related stress among HCWs involved in 2019-nCOVID with a prevalence ranging from 7.4 to 35%. Stress was more frequent in women, nurses, frontline workers, and in workers who experienced physical symptoms.

Our results about post-traumatic stress symptoms are consistent with those described in the two reviews above (16, 60), as occupational role, marital status, age and gender, quarantine, isolation emerged as robust risk factors for PTS symptoms. The study of Carmassi et al. (60) considered also stigma, previous psychiatric disorders, and being survivors of the same outbreak as risk factors for PTSD and PTS, but regrettably we did not gather such information. Carmassi et al. (60) suggested that HCWs caring for many SARS patients while working in high-risk units were less distressed, suggesting that non-frontline HCWs could show less psychological endurance. They also found that the perceived adequacy of training, a positive working organization, a sense of protection, clear communication of directives, and of precautionary measures represented protective factors against adverse outcomes of traumatic exposure. Support from family and friends as well as that from supervisors and colleagues was an important resilience factor against the development of PTSS.

Furthermore, psychological techniques for beating stress and trauma for the prevention of burnout may be helpful as well (59).

### Comparison With Other Italian Studies

As the constructs we have investigated, and particularly burnout, are influenced by the specific organization of the healthcare system, which shows many differences across nations, we will now discuss our findings in the light of the available Italian literature about the topic, to offer an overview of the Italian scenario.

We found many statistically significant results, which were quite consistent with those from previous studies, especially as regards depression, anxiety, and stress, while only few studies analyzed burnout in the HCWs population. Regrettably, in the current study, as well as in most of the available ones on this topic, there is no possibility of comparing burnout, post-traumatic stress symptoms, depression, and anxiety before the event, hence a precise measure of the real increase of these symptoms due to the pandemic situation cannot be achieved.

A study (17) conducted in Piedmont, as the current one, found GPs female, young or with a few years' work experience showing relevant anxiety and depressive symptoms, as we did even though in a different sample of HCWs. Our response rate was higher than in this study (12% of the contacted GPs vs. 37% of the present study).

Another study (18) on 73 nurses and 72 physicians through an anonymized online survey as part of a larger project investigating the psychological impact of 2019-nCOVID in Italian population revealed that nurses rated their health lower and reported to be more worried about contracting 2019-nCOVID compared to physicians; furthermore, they scored higher on measures of anxiety and post-traumatic stress symptoms. The results of the study mentioned above seem in line with the our own, as from the univariate analysis it emerged that nurses reported higher anxiety symptoms (BAI: OR nurses vs. medical doctors/physicians = 2.53, IC95% = 1.31–4.88) and higher risk to develop distress symptoms (IES: OR nurses vs. medical doctors/physicians = 1.67, IC95% = 1.16–2.40) than medical doctors/physicians.

Both depressive symptoms and PTSS were higher in HCWs working in COVID-19 wards; gender and marital status predicted depressive symptoms, while gender and age significantly predicted PTSS. Depressive symptoms were higher among females and HCWs not in a relationship, whereas higher levels of PTSS were observed among female and older HCWs.

Trumello et al. (20) investigated the psychological adjustment of 627 Italian HCWs during the peak of the 2019-nCOVID pandemic, with a web based survey sent through mainstream social media, including socio-demographic questions and three psychological test: contagion region Hamilton Depression Scale (HADS), Perceived Stress Scale (PSS-10), Professional Quality of Life (ProQOL-V). Lower levels of stress, burnout, secondary trauma, anxiety, and depression were observed among professionals not working with 2019-nCOVID patients.

In our research, we observed higher levels of D and EE (*p* < 0.01) among HCWs who changed working habits. Moreover, Trumello et al. (20) described higher levels of stress and burnout and lower levels of compassion satisfaction among HCWs of areas with elevate rates of contagion region (Lombardy, Piedmont, Emilia-Romagna, and Veneto, each one with more than 10,000 cases of infected patients during mid-April 2020), as in the present study, also conducted in an area with higher rates of 2019-nCOVID patients.

An online questionnaire spread via social networks between March 27 and March 31, 2020 (61) was completed by 1,379 HCWs. Younger age and female sex were associated with all investigated outcomes (PTSS, depressive symptoms, anxiety, perceived stress) except insomnia. Rossi et al. (61) reported that being a frontline HCWs and being GPs were associated with PTSS, nurses, and health care assistants were more likely to endorse severe insomnia, while in our study were reported higher levels of depression anxiety but lower D (*p* < 0.05). Being exposed to contagion was associated with symptoms of depression (OR: 1.54, 95% CI: 1.11–2.14, *p*-value 0.01).

In our study, it was observed that HCWs reporting health problems not related to 2019-nCOVID showed higher levels of depression, anxiety, stress and worst global mental health (*p* < 0.05). Moreover, HCWs who changed out-work habits reported higher levels of anxiety, depression and stress, worst global mental health, and also burnout symptoms, especially higher EE and D (*p*-value 0.05); finally, HCWs who changed jobs reported higher levels of stress and depression and higher EE and D, indicative of burnout (*p* < 0.05).

As mentioned above a 40-item online survey (47) was disseminated via social media to 527 Italian HCWs during the first 2019-nCOVID outbreak. Females and respondents working in high-risk sectors were more likely to rate psychological support as useful (OR: 1.78, CI 95% 1.14–2.78, *p*-value 0.012; OR: 2.02, 95% CI: 1.12–3.65, *p*-value 0.020, respectively) and workload as increased (OR: 0.38, 95% CI; 0.06–0.69, *p*-value 0.018; OR: 0.54, 95% CI: 0.16–0.92, *p*-value 0.005, respectively).

Another Italian study by Conti et al. (62) involved a large sample consisting of 933 HCWs during the 2019-nCOVID outbreak, and aimed to explore their mental health status and psychological care needs. HCWs female experienced higher levels of anxiety (*d* = 0.50) and somatization symptoms (*d* = 0.82) and reported higher need of psychological care (*p* < 0.001). HCWs aged <40 years-old reported higher levels of somatization, depression, anxiety, and post-traumatic symptoms (effects size *d* = [0.22–0.31]). As in our research, younger HCWs seem to suffer more from the psychological point of view, probably due to less years of work experience and less confidence with suffering and death; all these factors may contribute to the development of greater levels of burnout.

Overall, the available studies in Italy were consistent in the assessment of similar socio-demographic and work characteristics; similar psychological outcomes were also investigated, although with different tools. All the available studies were based on surveys, while none approached the topic of HCWs mental health with a structured clinical interview. Overall, the available studies are consistent in finding discomfort in HCWs, which is most frequent in women, those aged <30 years, nurses, in those who had to change their employment duties or in those who had a family member suffering from 2019-nCOVID. More specifically, in our study, we observed anxiety, stress, and depression, worst global mental health among females, who reported also burnout symptoms, especially higher EE (*p* < 0.05). In the current research we found higher levels of anxiety and burnout, especially D, among HCWs under the age of 30 years, and higher levels of burnout, with higher D and lower PA, among residents in training (*p* < 0.05). In our study, HCWs who changed jobs reported symptoms of stress and depression and higher EE and D (*p* < 0.05); furthermore, HCWs with loved ones affected by 2019-nCOVID reported higher levels of anxiety, stress and depression, and worst global mental health (*p* < 0.05).

It therefore seems important to define and to encourage the implementation of specific support approaches for HCWs, with particular attention to the more fragile populations (especially women, residents in training) who also seem to be those who tend to acknowledge more the importance of such an approach.

### Strengths and Limits

Among the strengths of our study there are sample size, the use of validated tools for mental health assessment, including burnout as well as anxiety, depression, post-traumatic symptoms, and overall mental health. Moreover, our sample included both frontline and not-frontline HCWs, recruited from the hospital as well as from extra-hospital settings, allowing a thorough understanding of the pandemic impact on HCWs at different levels. Furthermore, information was gathered about several socio-demographic, working habits-related, and pandemic-related variables.

Nonetheless, our research has some limitations which should be underscored. We gathered information only from a single-center in Piedmont, a high-risk, but restricted area, in Italy. This is a cross-sectional study, and according to this design, it is difficult to derive causal relationships. As all similar studies in this field, regrettably, objective data about previous psychiatric disorders were not available and we did not ask information about previous psychiatric history, which might bias the results we found. In our study we had no availability of previous measures of the psychological variables investigated, nonetheless, it is likely that, especially for burnout and perceived general health, scores worsened during the current pandemic.

Moreover, as in other similar studies, the psychological evaluation was conducted only though online self-report instruments, that are less precise and accurate than an evaluation by an experienced clinician with a clinical interview. Finally, the validated scale for the evaluation of stress symptoms (COVID-19 IES), specific to the current pandemic (37), has not been used as it was not yet available. Nonetheless, the further developments of this study include the follow-up of the sample adding COVID-specific measures.

## Conclusions

As suggested by previous works (14, 21, 56, 58, 63) the evidence we found points to the fact that burnout is a well-recognized psychological consequence of being a HCW during a pandemic emergency such as the current one.

The problematic levels of burnout and adverse psychological outcomes observed during the pandemic cannot be underestimated. The psychological well-being of HCWs is a priority to avoid reduced work performance. Therefore, it would be essential not only to monitor the level of psychological stress in these populations but also to develop support techniques for HCWs, including greater access to psychological support services, with the possibility to teach self-care strategies to better manage difficult situations in the workplace. Given the recurrence in autumn 2020 of a new pandemic peak, which has once again put a strain on the health system and HCWS, the importance of planning a follow-up of this population emerges. Further issues which deserve attention are the possible differences between frontline and not-frontline HCWs, as well as the possible risk and protective factors which can be identified both in the work environment and in the extra-work one. For this purpose we are planning the follow-up of the sample, also adding COVID-specific measures.

## Data Availability Statement

The raw data supporting the conclusions of this article will be made available by the authors, without undue reservation.

## Ethics Statement

The studies involving human participants were reviewed and approved by Comitato Etico Interaziendale di Novara, Protocollo 534/CE, Studio n. CE 82/20, approved on May 11th, 2020. The patients/participants provided their written informed consent to participate in this study.

## Author Contributions

PZ and CGr designed the study. DM, CGu, LL, MP, MR, and EG contributed to disseminating the survey. DA performed the statistical analyses. PZ, CGr, and EG drafted the manuscript. All authors revised the manuscript and contributed with relevant intellectual content.

## Conflict of Interest

The authors declare that the research was conducted in the absence of any commercial or financial relationships that could be construed as a potential conflict of interest.

## References

[1] Wei E Segall J Villanueva Y Dang LB Gasca VI Gonzalez MP. Coping with trauma, celebrating life: reinventing patient and staff support during the covid-19 pandemic. Health Aff. (2020) 39:1597–600. 10.1377/hlthaff.2020.00929 32673086

[2] Salazar de Pablo G Vaquerizo-Serrano J Catalan A Arango C Moreno C Ferre F. Impact of coronavirus syndromes on physical and mental health of health care workers: systematic review and meta-analysis. J Affect Disord. (2020)275:48–57. 10.1016/j.jad.2020.06.022 32658823 PMC7314697

[3] Serrano-Ripoll MJ Meneses-Echavez JF Ricci-Cabello I Fraile-Navarro D Fiol-deRoque MA Pastor-Moreno G. Impact of viral epidemic outbreaks on mental health of healthcare workers: a rapid systematic review and meta-analysis. J Affect Disord. (2020) 277:347–57. 10.1016/j.jad.2020.08.034 32861835 PMC7443314

[4] Shreffler J Petrey J Huecker M. The impact of COVID-19 on healthcare worker wellness: a scoping review. West J Emerg Med. (2020) 21:1059–66. 10.5811/westjem.2020.7.48684 32970555 PMC7514392

[5] Preti E Di Mattei V Perego G Ferrari F Mazzetti M Taranto P. The psychological impact of epidemic and pandemic outbreaks on healthcare workers: rapid review of the evidence. Curr Psychiatry Rep. (2020) 22:43. 10.1007/s11920-020-01166-z 32651717 PMC7350408

[6] Pappa S Ntella V Giannakas T Giannakoulis VG Papoutsi E Katsaounou P. Prevalence of depression, anxiety, and insomnia among healthcare workers during the COVID-19 pandemic: a systematic review and meta-analysis. Brain Behav Immun. (2020) 88:901–7. 10.1016/j.bbi.2020.05.026 32437915 PMC7206431

[7] Sharifi M Asadi-Pooya AA Mousavi-Roknabadi RS. Burnout among healthcare providers of COVID-19; a systematic review of epidemiology and recommendations. Arch Acad Emerg Med. (2020) 9:e7. 10.22037/aaem.v9i1.1004 33490964 PMC7812159

[8] Maslach C Leiter MP. Understanding the burnout experience: recent research and its implications for psychiatry. World Psychiatry Off J World Psychiatr Assoc WPA. (2016) 15:103–11. 10.1002/wps.20311 27265691 PMC4911781

[9] Hu D Kong Y Li W Han Q Zhang X Zhu LX. Frontline nurses' burnout, anxiety, depression, and fear statuses and their associated factors during the COVID-19 outbreak in Wuhan, China: a large-scale cross-sectional study. EClinicalMedicine. (2020) 24:100424. 10.1016/j.eclinm.2020.100424 32766539 PMC7320259

[10] Sung C-W Chen C-H Fan C-Y Su F Chang J-H Hung C-C. Burnout in medical staffs during a coronavirus disease (COVID-19) pandemic. SSRN Electron J. (2020). 10.2139/ssrn.3594567

[11] Sahin T Aslaner H Eker OO Gokcek MB Dogan M. Effect of COVID-19 pandemic on anxiety and burnout levels in emergency healthcare workers: a questionnaire study. Res Square. (2020) 10.21203/rs.3.rs-32073/v1

[12] Kannampallil TG Goss CW Evanoff BA Strickland JR McAlister RP Duncan J. Exposure to COVID-19 patients increases physician trainee stress and burnout. PLoS ONE. (2020) 15:e0237301. 10.1371/journal.pone.0237301 32760131 PMC7410237

[13] Wu Y Wang J Luo C Hu S Lin X Anderson AE. A comparison of burnout frequency among oncology physicians and nurses working on the frontline and usual wards during the COVID-19 epidemic in Wuhan, China. J Pain Symptom Manage. (2020) 60:e60–5. 10.1016/j.jpainsymman.2020.04.008 32283221 PMC7151285

[14] Dimitriu MCT Pantea-Stoian A Smaranda AC Nica AA Carap AC Constantin VD. Burnout syndrome in Romanian medical residents in time of the COVID-19 pandemic. Med Hypotheses. (2020) 144:109972. 10.1016/j.mehy.2020.109972 32531540 PMC7276114

[15] Xiang YT Jin Y Wang Y Zhang Q Zhang L Cheung T. Tribute to health workers in China: a group of respectable population during the outbreak of the COVID-19. Int J Biol Sci. (2020) 16:1739–40. 10.7150/ijbs.45135 32226292 PMC7098026

[16] Benfante A Di Tella M Romeo A Castelli L. Traumatic stress in healthcare workers during COVID-19 pandemic: a review of the immediate impact [Internet]. Front Psychol. (2020) 11:2816. 10.3389/fpsyg.2020.569935 PMC7645025 33192854

[17] Castelli L Di Tella M Benfante A Taraschi A Bonagura G Pizzini A. The psychological impact of COVID-19 on general practitioners in Piedmont, Italy. J Affect Disord. (2021) 281:244–6. 10.1016/j.jad.2020.12.008 33338842 PMC7835105

[18] Di Tella M Benfante A Castelli L Romeo A. Anxiety, depression, and posttraumatic stress in nurses during the COVID-19 outbreak. Intensive Crit Care Nurs. (2021) 103014. 10.1016/j.iccn.2021.103014 33589343 PMC7857052

[19] Giusti EM Pedroli E D'Aniello GE Stramba Badiale C Pietrabissa G Manna C. The psychological impact of the COVID-19 outbreak on health professionals: a cross-sectional study. Front Psychol. (2020) 11:1684. 10.3389/fpsyg.2020.01684 32754102 PMC7366071

[20] Trumello C Bramanti SM Ballarotto G Candelori C Cerniglia L Cimino S. Psychological adjustment of healthcare workers in Italy during the COVID-19 Pandemic: differences in stress, anxiety, depression, burnout, secondary trauma, and compassion satisfaction between frontline and non-frontline professionals. Int J Environ Res Public Health. (2020) 17:8358. 10.3390/ijerph17228358 33198084 PMC7696387

[21] Barello S Palamenghi L Graffigna G. Burnout and somatic symptoms among frontline healthcare professionals at the peak of the Italian COVID-19 pandemic. Psychiatry Res. (2020) 290:113129. 10.1016/j.psychres.2020.113129 32485487 PMC7255285

[22] Buselli R Corsi M Baldanzi S Chiumiento M Lupo ED Dell'oste V. Professional quality of life and mental health outcomes among health care workers exposed to SARS-CoV-2 (COVID-19). Int J Environ Res Public Health. (2020) 17:1–12. 10.3390/ijerph17176180 32858810 PMC7504107

[23] Franza F Basta R Pellegrino F Solomita B Fasano V. The role of fatigue of compassion, burnout and hopelessness in healthcare: experience in the time of Covid-19 outbreak. Psychiatr Danub. (2020) 32(Suppl 1):S10–4. 32890354

[24] Vagni M Maiorano T Giostra V Pajardi D. Coping with COVID-19: emergency stress, secondary trauma and self-efficacy in healthcare and emergency workers in Italy. Front Psychol. (2020) 11:566912. 10.3389/fpsyg.2020.566912 33013603 PMC7494735

[25] Maiorano T Vagni M Giostra V Pajardi D. COVID-19: risk factors and protective role of resilience and coping strategies for emergency stress and secondary trauma in medical staff and emergency workers—an online-based inquiry. Sustain Switz. (2020) 12:1–18. 10.3390/su12219004

[26] Nezafat Maldonado BM Collins J Blundell HJ Singh L. Engaging the vulnerable: a rapid review of public health communication aimed at migrants during the COVID-19 pandemic in Europe. J Migr Health. (2020) 1:100004. 10.1016/j.jmh.2020.100004 33447830 PMC7661962

[27] Maslach C Leiter MP. Early predictors of job burnout and engagement. J Appl Psychol. (2008) 93:498–512. 10.1037/0021-9010.93.3.498 18457483

[28] Sirigatti S Stefanile C. MBI - Maslach Burnout Inventory. Adattamento e taratura per l'Italia. Firenze: OS Organizzazioni Speciali (1993).

[29] Giorgi G Leon-Perez JM Castiello D'Antonio A Fiz Perez FJ Arcangeli G Cupelli V. The General Health Questionaire (GHQ-12) in a sample of Italian workers: mental health at individual and organizational level. World J Med Sci. (2014) 11:47–56. 10.3389/fpsyg.2016.00060

[30] Fraccaroli F Depolo M Sarchielli G. L'uso del General Health Questionnaire di Goldberg in una ricerca su giovani disoccupati [The use of Goldberg's General Health Questionnaire in a study of unemployed young people]. Giunti Organ Spec. (1991) 197:13–9.

[31] Goldberg DP Hillier VF. A scaled version of the General Health Questionnaire. Psychol Med. (1979) 9:139–45. 10.1017/S0033291700021644 424481

[32] Qin M Vlachantoni A Evandrou M Falkingham J. General Health Questionnaire-12 reliability, factor structure, and external validity among older adults in India. Indian J Psychiatry. (2018) 60:56. 10.4103/psychiatry.IndianJPsychiatry_112_17 29736063 PMC5914263

[33] Horowitz M Wilner N Alvarez W. Impact of event scale: a measure of subjective stress. Psychosom Med. (1979) 41:209–18. 10.1097/00006842-197905000-00004 472086

[34] Briere J. Psychological assessment of adult posttraumatic states. Washington, DC: American Psychological Association (1997) xv, 251. p. 10.1037/10267-000

[35] Corcoran K Fischer J. Measures for Clinical Practice: A Sourcebook, Vol. 2 Adults (3rd ed.). New York, NY: Free Press (1994).

[36] Pietrantonio F De Gennaro L Di Paolo MC Solano L. The impact of event scale: validation of an Italian version. J Psychosom Res. (2003) 55:389–93. 10.1016/S0022-3999(02)00638-4 14507552

[37] Vanaken L Scheveneels S Belmans E Hermans D. Validation of the Impact of Event Scale With Modifications for COVID-19 (IES-COVID19). Front Psychiatry. (2020) 11:738. 10.3389/fpsyt.2020.00738 32848918 PMC7399230

[38] Fydrich T Dowdall D Chambless DL. Reliability and validity of the beck anxiety inventory. J Anxiety Disord. (1992) 6:55–61. 10.1016/0887-6185(92)90026-433114144

[39] Beck AT Epstein N Brown G Steer RA. An inventory for measuring clinical anxiety: psychometric properties. J Consult Clin Psychol. (1988) 56:893–7. 10.1037/0022-006X.56.6.893 3204199

[40] Sica C Ghisi M. The Italian versions of the Beck Anxiety Inventory and the Beck Depression Inventory-II: psychometric properties and discriminant power. In: Lange MA, editor. Leading-Edge Psychological Tests and Testing Research. Hauppauge, NY: Nova Science Publishers, Inc. (2007) p. 27–50.

[41] Beck AT Steer RA Carbin MG. Psychometric properties of the Beck Depression Inventory: Twenty-five years of evaluation. Clin Psychol Rev. (1988) 8:77–100. 10.1016/0272-7358(88)90050-5

[42] Toledano-Toledano F Contreras-Valdez JA. Validity and reliability of the Beck Depression Inventory II (BDI-II) in family caregivers of children with chronic diseases. PLoS ONE. (2018) 13:e0206917. 10.1371/journal.pone.0206917 30485299 PMC6261561

[43] Harrell FEJ. rms: Regression Modeling Strategies. R package version 4.1-3 (2014).

[44] Team RC. R: A Language and Environment for Statistical Computing. Vienna: R Foundation for Statistical Computing (2018).

[45] Lu W Wang H Lin Y Li L. Psychological status of medical workforce during the COVID-19 pandemic: a cross-sectional study. Psychiatry Res. (2020) 288:112936. 10.1016/j.psychres.2020.112936 32276196 PMC7195354

[46] Tan BYQ Chew NWS Lee GKH Jing M Goh Y Yeo LLL. Psychological impact of the COVID-19 pandemic on health care workers in Singapore [Internet]. Ann Intern Med. (2020) 173:317–20. 10.7326/M20-1083 32251513 PMC7143149

[47] Felice C Di Tanna GL Zanus G Grossi U. Impact of COVID-19 outbreak on healthcare workers in Italy: results from a National E-Survey. J Community Health. (2020) 45:675–83. 10.1007/s10900-020-00845-5 32440724 PMC7242177

[48] Yuan S Liao Z Huang H Jiang B Zhang X Wang Y. Comparison of the indicators of psychological stress in the population of hubei province and non-endemic provinces in China during two weeks during the coronavirus disease (2019). (COVID-19) outbreak in february 2020. Med Sci Monit. (2020). 26:e923767–1. 10.12659/MSM.923767 32294078 PMC7177041

[49] Zhu J Sun L Zhang L Wang H Fan A Yang B. Prevalence and influencing factors of anxiety and depression symptoms in the first-line medical staff fighting against COVID-19 in Gansu. Front Psychiatry. (2020) 11:386. 10.3389/fpsyt.2020.00386 32411034 PMC7202136

[50] Cai H Tu B Ma J Chen L Fu L Jiang Y. Psychological impact and coping strategies of frontline medical staff in Hunan between January and March 2020 during the outbreak of coronavirus disease (2019). (COVID) in Hubei, China. Med Sci Monit. (2020) 26:e924171–1. 10.12659/MSM.924171 32291383 PMC7177038

[51] Muller AE Hafstad EV Himmels JPW Smedslund G Flottorp S Stensland SØ. The mental health impact of the covid-19 pandemic on healthcare workers, and interventions to help them: a rapid systematic review [Internet]. Psychiatry Res. (2020) 293:113441. 10.1016/j.psychres.2020.113441 32898840 PMC7462563

[52] Zerbini G Ebigbo A Reicherts P Kunz M Messman H. Psychosocial burden of healthcare professionals in times of covid-19 – a survey conducted at the university hospital augsburg. Ger Med Sci. (2020) 18:Doc05. 10.3205/000281 32595421 PMC7314868

[53] Greenberg N. Mental health of health-care workers in the COVID-19 era [Internet]. Nat Rev Nephro. (2020) 16:425–6. 10.1038/s41581-020-0314-5 32561871 PMC7304244

[54] Spoorthy MS. Mental health problems faced by healthcare workers due to the COVID-19 pandemic–A review [Internet]. Asian J Psychiatry. (2020) 51:102119. 10.1016/j.ajp.2020.102119 PMC7175897 32339895

[55] Azoulay E De Waele J Ferrer R Staudinger T Borkowska M Povoa P. Symptoms of burnout in intensive care unit specialists facing the COVID-19 outbreak. Ann Intensive Care. (2020) 10:110. 10.1186/s13613-020-00722-3 32770449 PMC7414284

[56] Luceño-Moreno L Talavera-Velasco B García-Albuerne Y Martín-García J. Symptoms of posttraumatic stress, anxiety, depression, levels of resilience and burnout in spanish health personnel during the COVID-19 pandemic. Int J Environ Res Public Health. (2020) 17:1–29. 10.3390/ijerph17155514 32751624 PMC7432016

[57] Padula MS Ilari G. Baraldi S Guaraldi GP Ferretti E Musiani V. Il burnout nella Medicina Generale: personalità del medico e personalità del paziente. Riv Della Soc Ital Med Gen. (2008) 4:42–7.

[58] Asai M Morita T Akechi T Sugawara Y Fujimori M Akizuki N. Burnout and psychiatric morbility among physicians engaged in end-to-life care for cancer patients: a cross-sectional nationwide survey in Japan. Psychooncology. (2007) 16:421–8. 10.1002/pon.1066 16929464

[59] Vizheh M Qorbani M Arzaghi SM Muhidin S Javanmard Z Esmaeili M. The mental health of healthcare workers in the COVID-19 pandemic: a systematic review [Internet]. J Diabetes Metab Disord. (2020) 19:1–12. 10.1007/s40200-020-00643-9 PMC7586202 33134211

[60] Carmassi C Foghi C Dell'Oste V Cordone A Bertelloni CA Bui E. PTSD symptoms in healthcare workers facing the three coronavirus outbreaks: what can we expect after the COVID-19 pandemic. Psychiatry Res. (2020) 292:113312. 10.1016/j.psychres.2020.113312 32717711 PMC7370915

[61] Rossi R Socci V Pacitti F Di Lorenzo G Di Marco A Siracusano A. Mental health outcomes among frontline and second-line health care workers during the coronavirus disease 2019. (COVID-19) Pandemic in Italy. JAMA Netw Open. (2020) 3:e2010185. 10.1001/jamanetworkopen.2020.10185 32463467 PMC7256664

[62] Conti C Fontanesi L Lanzara R Rosa I Porcelli P. Fragile heroes. the psychological impact of the COVID-19 pandemic on health-care workers in Italy. PLOS ONE. (2020) 15:e0242538. 10.1371/journal.pone.0242538 33206714 PMC7673566

[63] Raudenská J Steinerová V Javurková A Urits I Kaye AD Viswanath O. Occupational burnout syndrome and post-traumatic stress among healthcare professionals during the novel coronavirus disease 2019. (COVID-19) pandemic. Best Pract Res Clin Anaesthesiol. (2020) 34:553–60. 10.1016/j.bpa.2020.07.008 33004166 PMC7367798

