# Supplementary material for: The Mental Health Impact of 2019-nCOVID on Healthcare Workers From North-Eastern Piedmont, Italy. Focus on Burnout
Source: Front Public Health. 2021 May 11;9:667379. doi: 10.3389/fpubh.2021.667379 (PMC8144493; doi:10.3389/fpubh.2021.667379)
Supplement: Supplementary file 1 [file Data_Sheet_1.docx]

| **Field Label** | **Choices, Calculations, OR Slider Labels** | **Field Note** |
| --- | --- | --- |
| Record ID |  |  |
| Email address |  |  |
| I consent to the participation of the study under the above conditions and  I declare that the objectives of the survey have been clearly explained | 1, Yes |  |
| Age |  |  |
| Children | 1, Male \| 2, Female |  |
| Civil status | 1, Single/Unmarried \| 2, Married \| 3, Divorced \| 4, Cohabitant \| 5, Widower \| 6, In a romantic relationship |  |
| Children | 1, Yes \| 0, No |  |
| Was it positive for COVID-19? | 1, Yes \| 0, No |  |
| Have you had covid-19-related symptoms? | 1, Yes \| 0, No |  |
| Have you had any non-COVID-19-related health issues? | 1, Yes \| 0, No |  |
| Has anyone dear to you tested positive for COVID-19? | 1, Yes \| 0, No |  |
| Following the COVID-19 pandemic, did you change its habits  Family? | 1, Yes \| 0, No |  |
| Did you change your family habits for fear of infecting familiars? | 1, Yes \| 0, No |  |
| Working category | 1, Medical doctor/Physicians 2, Residents in trainings 3, Nurses 4, Others (such as psychologists, social workers-health, psychological, radiological and laboratory technicians, educators) |  |
| Has your job been changed during the COVID-19 emergency? | 1, Yes \| 0, No |  |
